# Secondary cross-sectional analysis of smoking and drinking factors among older Korean men: A 13-year national survey

**DOI:** 10.18332/tid/211500

**Published:** 2025-11-21

**Authors:** Jimin Lee, Youngmee Kim, Won-Kyung Cho

**Affiliations:** 1 Seoul Metropolitan Government-Seoul National University, Boramae Medical Center, Seoul, Republic of Korea; 2 Red Cross College of Nursing, Chung-Ang University, Seoul, Republic of Korea; 3 International Healthcare Center, Asan Medical Center, University of Ulsan, Seoul, Republic of Korea; 4 Pulmonary and Critical Care Medicine, Asan Medical Center, University of Ulsan College of Medicine, Seoul, Republic of Korea

**Keywords:** smoking, drinking, older adult, Korean, men

## Abstract

**INTRODUCTION:**

Given the shared risk profiles and interdependence of smoking and drinking behaviors, this study aimed to examine factors associated with smoking and alcohol consumption among older adult Korean men.

**METHODS:**

This was a secondary cross-sectional analysis of data pooled from the Korea National Health and Nutrition Examination Survey (KNHANES) conducted between 2007 and 2019, encompassing 7259 men aged ≥65 years. The participants were divided into the non-smoking/non-drinking, smoking/non-drinking, non-smoking/drinking, and smoking/drinking groups, and the sociodemographic and various health-related data collected via questionnaires and blood tests were used for multinomial logistic regression.

**RESULTS:**

When examining factors associated with each group using the non-smoking/non-drinking group as the reference group, several associated factors were identified. For example, not participating in regular exercise (AOR=1.31; 95% CI: 1.06–1.61; p=0.011), perceived health status as poor/very poor (AOR=1.56; 95% CI: 1.21–2.00; p<0.001), and skipping breakfast (AOR=1.90; 95% CI: 1.22–2.98; p=0.005) were some of the factors positively associated with the smoking/non-drinking group. Conversely BMI ≥25 (AOR=1.44; 95% CI: 1.12–1.83; p=0.004), elevated triglyceride levels (AOR=1.03; 95% CI: 1.03–1.05; p<0.001), and more daily fat intake (AOR=1.20; 95% CI: 1.03–1.40; p=0.019) were positively associated with the non-smoking/drinking group. Higher triglyceride levels (AOR=1.03; 95% CI: 1.01–1.05; p<0.001), depressive mood (AOR=2.10; 95% CI: 1.20–3.67; p=0.009), and more daily fat intake (AOR=1. 27; 95% CI: 1.07–1.51; p=0.007) were positively associated with the smoking/drinking group.

**CONCLUSIONS:**

Metabolic changes, such as higher triglyceride levels, were more common in the drinking groups, whereas negative emotions were more prevalent among smokers. These findings may highlight the need for targeted interventions to promote healthier lifestyles among older adults; however, further research is necessary to revalidate our study findings.

## INTRODUCTION

Unhealthy lifestyle habits such as smoking and alcohol consumption are among the most common behaviors that negatively impact health^1,2^. Excessive smoking and alcohol consumption may lead to addiction, and the prevalence thereof remains a major public health concern both globally and in The Republic of Korea^1,2^. These habits often co-occur, with evidence suggesting a strong interrelationship between the two behaviors. One longitudinal study found that smokers who drank moderately had 1.54 times the odds of continuing smoking at 1 month, and heavy drinkers had 2.59 times the odds of continuing smoking compared to non-drinkers. After 7 months, heavy drinkers still had a 2.32 times higher likelihood of still smoking^3^. Behaviorally, smokers who drink alcohol have reported smoking more while drinking, as well as smoking more when in social settings such as bars or clubs^4^. Furthermore, chronic alcohol use speeds up nicotine metabolism, making withdrawal sharper, and smoking more likely to relapse^5^.

Despite the decline in global smoking prevalence, the burden of tobacco use remains a significant public health concern^6^. As of 2022, the global adult smoking rate was approximately 21.7%^6^, with South Korea reporting an adult prevalence of approximately 18%^6,7^. These figures indicate that a substantial proportion of the adult population continue to smoke, underscoring the need for effective intervention strategies. In this context, it is posited that regulating associated behavioral risk factors, such as alcohol intake, could serve as a complementary strategy to promote smoking cessation.

Ageing is associated with physiological changes and increased vulnerability to chronic diseases. As the population of older adults grows, understanding the health behaviors of this age group becomes increasingly important. Smoking and drinking in older adults are particularly concerning due to their cumulative health impacts and potential interactions with age-related conditions and medications^8-10^. Patterns of tobacco and alcohol use may differ significantly in older adults compared to younger populations. These patterns are influenced by various sociodemographic and health-related factors^11-13^. Older adults experience more pronounced effects from alcohol due to changes in body composition and metabolism, such as decreased lean body mass and water content and a slower metabolic rate^14^. Older adults who smoke are more likely to be nicotine-dependent and face challenges in quitting smoking^15^. Smoking can lead to social isolation and disengagement, while drinking can be associated with living alone^16^.

Given the shared risk profiles and interdependence of smoking and drinking behaviors, it is important to examine their combined effects and associated characteristics in older adults. Identifying the factors associated with these behaviors may offer valuable insights to develop targeted interventions to promote healthier lifestyles, leading to smoking cessation among older adults. We, therefore, aimed to examine the factors associated with smoking and alcohol consumption among Korean men aged ≥65 years using data from the Korea National Health and Nutrition Examination Survey (KNHANES)^17^.

## METHODS

### Study design and ethical considerations

This was a secondary cross-sectional analysis of data pooled from the Korea National Health and Nutrition Examination Survey (KNHANES) conducted between 2007 and 2019, encompassing 7259 men aged ≥65 years. The KNHANES, a nationwide population-based survey designed to assess the overall health, lifestyle behaviors, and dietary habits of Korean residents, conducted annually by the Korea Disease Control and Prevention Agency (KDCA)^17^. The survey consisted of three core sections: a health interview, health examination, and nutrition survey. Trained healthcare professionals and interviewers conducted the interviews and examinations in specially equipped mobile examination centers. Data from the KNHANES were collected using a stratified multistage cluster probability sampling strategy to ensure that the entire Korean population was accurately represented. The analysis incorporated the complex sampling of the KNHANES by applying the survey procedure with individual weights, considering the variance estimation strata (KSTRATA), cluster sampling unit, and integrated sampling weights. Data were obtained through the official registration process on the KNHANES website^17^. All participants provided written informed consent prior to participation, in accordance with the principles outlined in the Declaration of Helsinki. The KDCA Institutional Review Board meticulously reviewed and approved the KNHANES survey (approval numbers: 2007-02CON-04-P, 2008-04EXP-01-C, 2009-01CON-03-2C, 2010-02CON-21-C, 2011-02CON-06-C, 2012-01EXP-01-2C, 2013-07CON-03-4C, 2013-12EXP-03-5C, 2018-01-03-P-A, and 2018-01-03-C-A).

### Participants

**Figure 1:**
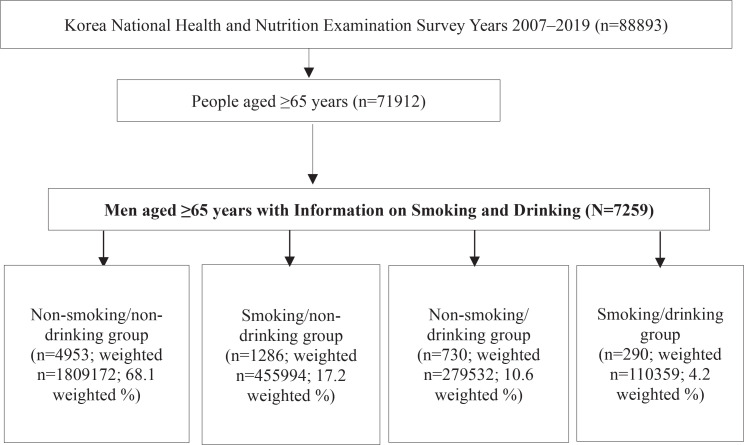
Flow diagram of participant selection and the number of participants in each group

Of the 88893 individuals screened, 7259 men aged ≥65 years with information on smoking and drinking habits were enrolled. The participants were then categorized into four groups according to their smoking and drinking status: non-smoking and non-drinking (non-smokers/non-drinkers), smoking and non-drinking (smokers/non-drinkers), non-smoking and drinking (non-smokers/drinkers), and smoking and drinking (smokers/drinkers), with 4953 participants (weighted n=1809172; 68.1 weighted %), 1286 participants (weighted n=455994; 17.2 weighted %), 730 participants (weighted n=279532; 10.6 weighted %), and 290 participants (weighted n=110359; 4.2 weighted %) in each group, respectively (Figure 1).

### Definitions and variables

Smoking was defined as having smoked at least 100 cigarettes in one’s lifetime and currently smoking^18^. Drinking was operationally defined as consuming alcohol at least twice a week, regardless of the type, over the course of the past year and drinking more than five drinks at a time. Regular exercise was operationally defined as participation in either vigorous or moderate physical activity. Vigorous physical activity included high-intensity exercises such as running, performed for at least 1.25 hours per week. Moderate physical activity included moderate-intensity exercises, such as yoga, performed for at least 2.5 hours per week. Activity limitation was defined as difficulty performing specific tasks due to physical or mental health conditions.

Additional variables included perceived health status, assessed as an individual’s subjective evaluation of their overall health and categorized as very good/good, fair, or poor/very poor; and perceived psychological stress, defined as experiencing moderate to severe stress on a daily basis. Depressive mood was defined as experiencing feelings of depression for at least 2 consecutive weeks. A history of trauma was defined as having experienced at least one incident that required hospitalization and/or emergency room treatment due to an accident or intoxication in the past year. Hypertension was defined as systolic blood pressure ≥140 mmHg, a diastolic blood pressure ≥90 mmHg, or use of antihypertensive medication. Diabetes mellitus was defined as a fasting plasma glucose level ≥126 mg/dL, use of antidiabetic medication or insulin, a physician’s diagnosis of diabetes mellitus, or a glycated hemoglobin (HbA1c) level ≥6.5%. Anemia was defined as a hemoglobin level of <13 g/dL in men. Cardiovascular diseases were defined as a medical diagnosis of angina or myocardial infarction, while musculoskeletal diseases were defined as a medical diagnosis of arthritis, osteoarthritis, rheumatoid arthritis, or osteoporosis. Variables for other comorbidities were assessed based on a history of diagnosis and treatment, as well as current ongoing treatment. All the variable definitions in this study were based on the KNHANES questionnaire^17^.

### Statistical analysis

All data were analyzed using SAS version 9.4 (SAS Institute Inc., Cary, NC). Statistical significance was determined by a p<0.05, or a 95% confidence interval (CI) that did not span 1.0. The data are presented as mean ± standard error (SE) for continuous variables and as proportions ± SE for categorical variables.

Differences in sociodemographic and clinical characteristics, health-related characteristics, perceived health status, and nutritional status were evaluated using analysis of variance and the Rao-Scott chi-squared test for variables according to participant smoking and alcohol consumption status. For example, analysis of variance (ANOVA) was applied to compare the means of quantitative variables, such as age or blood pressure, while the Rao-Scott chi-squared test was employed to assess differences or associations in the distribution of qualitative variables, such as yes/no response, among these groups. To identify the factors associated with smoking and alcohol consumption, a multinomial logistic regression analysis was conducted. Variables with p<0.1 in the univariate analyses, including sociodemographic, clinical, perceived health status, health-related and nutritional characteristics, were adjusted for in the multivariable model. The smoking/non-drinking, non-smoking/drinking, and smoking/drinking groups were compared using the non-smoking/non-drinking group as a reference. When both continuous and categorical forms of a variable met the inclusion criteria, or when multicollinearity was detected, only one representative form was selected to avoid redundancy. Multicollinearity between variables was assessed, and variables with a variance inflation factor (VIF) >10 were excluded from the analysis. For example, WC was not included in the analysis because of multicollinearity between body mass index (BMI) and waist circumference (WC). Consequently, the outcomes of the analysis were smoking and drinking status, while the predictors, considered as potential confounders, included the following variables: age, marital status, cohabitation, residence, employment status, household income, types of health insurance, education level, systolic blood pressure, body mass index ≥25 kg/m^2^, total cholesterol, high-density lipoprotein cholesterol, triglycerides, hemoglobin, fasting blood glucose, hypertension, cardiovascular disease, cancer, anemia, stroke, activity limitation, regular exercise, perceived health status, perceived stress, depressive mood, skipping meals (breakfast, lunch, and dinner), daily intake of macronutrients and minerals (carbohydrates, protein, fat, calcium, iron, and sodium ≥2000 mg), and energy contribution of carbohydrates, and fat. The weights used in this study incorporate non-response errors (missing data) from non-participants; therefore, no separate handling of missing data was performed.

## RESULTS

### Changes in prevalence of smoking and drinking

The participants were then categorized into four groups according to their smoking and drinking status: non-smoking and non-drinking (non-smokers/non-drinkers), smoking and non-drinking (smokers/non-drinkers), non-smoking and drinking (non-smokers/drinkers), and smoking and drinking (smokers/drinkers), with 4953 participants (weighted n=1809172; 68.1 weighted %), 1286 participants (weighted n=455994; 17.2 weighted %), 730 participants (weighted n=279532; 10.6 weighted %), and 290 participants (weighted n=110359; 4.2 weighted %) in each group, respectively (Figure 1). The mean age for each group was as follows: non-smoking and non-drinking group, 72.6 ± 0.09 years; smoking and non-drinking group, 72.0 ± 0.16 years; non-smoking and drinking group, 71.0 ± 0.18 years and smoking and drinking group, 70 ± 0.30 years [mean ± standard error (SE)].

**Figure 2:**
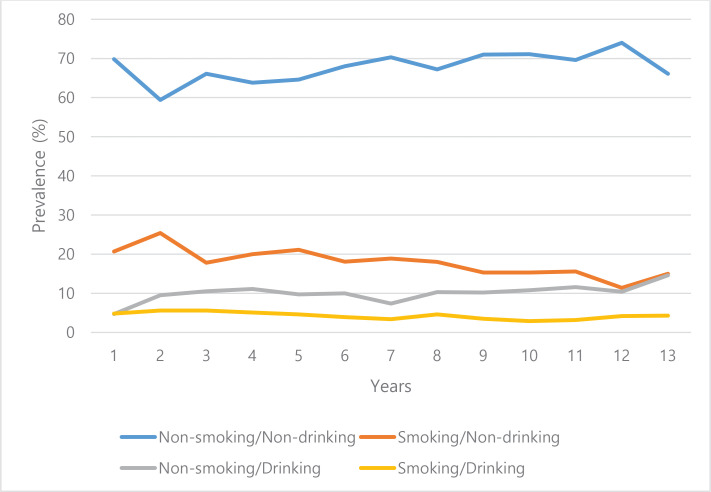
Prevalence of smoking and drinking among male participants aged ≥65 years, KNHANES 2007–2019

Figure 2 illustrates the prevalence of smoking and/or drinking for 13 years across the four groups. Over the 13-year study period, all groups maintained a stable trend without any notable changes. The non-smoking/non-drinking group consistently accounted for the largest proportion, whereas the smoking/non-drinking and non-smoking/drinking groups showed an increasing trend in prevalence in recent years.

### Sociodemographic, clinical, and health behaviors

Table 1 presents sociodemographic characteristics according to smoking and drinking status. The non-smoking/non-drinking group had the highest proportion of participants who were married, lived with family, and were university graduates. Regarding household income, the non-smoking/drinking group had the highest proportion in the 1st quartile (highest income). The proportion of people living in the city, employed, and elementary graduates was highest in the smoking/drinking group compared to the other groups.

**Table 1:** Sociodemographic characteristics of male participants, aged ≥65 years, according to smoking and drinking status, KNHANES 2007–2019

*Characteristics*	*Non-smoking/* *non-drinking* *(1)*	*Smoking/* *non-drinking* *(2)*	*Non-smoking/* *drinking* *(3)*	*Smoking/* *drinking* *(4)*	*p*	*p by multiple comparison*					
						*(1) vs* *(2)*	*(1) vs* *(3)*	*(1) vs* *(4)*	*(2) vs* *(3)*	*(2) vs* *(4)*	*(3) vs* *(4)*
**Total**, n (weighted n)	4953 (1809172)	1286 (455994)	730 (279532)	290 (110359)							
**Age** (years)					<0.001	0.002	<0.001	<0.001	<0.001	<0.001	0.034
65–74	62.2 (0.83)	67.8 (1.56)	76.9 (1.73)	83.8 (2.52)							
≥75	37.8 (0.83)	32.2 (1.56)	23.1 (1.73)	16.2 (2.52)							
Mean	72.6 (0.09)	72.0 (0.16)	71.0 (0.18)	70.0 (0.30)							
**Marital status**					<0.001	<0.001	0.686	0.039	0.009	0.652	0.138
Married	90.5 (0.50)	85.4 (1.22)	90.0 (1.24)	86.6 (2.20)							
Separated/divorced/widowed	9.5 (0.50)	14.6 (1.22)	10.0 (1.24)	13.4 (2.20)							
**Cohabitation**					<0.001	<0.001	0.282	<0.001	0.045	0.408	0.025
Living alone	7.7 (0.41)	12.0 (0.99)	9.0 (1.15)	13.8 (2.09)							
Living with family or others	92.3 (0.41)	88.0 (0.99)	91.0 (1.15)	86.2 (2.09)							
**Residence**					0.005	0.001	0.729	0.410	0.051	0.011	0.339
Urban	74.7 (1.24)	69.3 (1.89)	74.0 (2.20)	76.9 (2.74)							
Rural	25.3 (1.24)	30.7 (1.89)	26.0 (2.20)	23.1 (2.74)							
**Currently employed**					<0.001	<0.001	0.009	0.051	0.410	0.788	0.775
Yes	37.3 (0.90)	45.2 (1.81)	43.0 (2.14)	44.1 (3.45)							
No	62.7 (0.90)	54.8 (1.81)	57.0 (2.14)	55.9 (3.45)							
**Household income** (quartiles)					<0.001	0.000	0.303	0.003	0.042	0.482	0.016
1st (highest)	27.6 (0.82)	22.2 (1.48)	27.9 (2.06)	21.2 (2.76)							
2nd	26.1 (0.75)	23.8 (1.41)	22.7 (1.86)	22.8 (3.01)							
3rd	23.5 (0.69)	25.6 (1.43)	26.5 (2.00)	22.4 (2.81)							
4th (lowest)	22.7 (0.73)	28.4 (1.49)	22.9 (1.81)	33.6 (3.23)							
**Types of health insurance**					<0.001	<0.001	0.372	0.012	0.004	0.543	0.009
National health insurance	96.1 (0.34)	93.6 (0.77)	97.0 (0.80)	92.4 (1.95)							
Government medical aids for low income	3.9 (0.34)	6.4 (0.77)	3.0 (0.80)	7.6 (1.95)							
**Education level**					<0.001	<0.001	0.749	0.007	0.013	0.870	0.078
≤ Elementary school	41.3 (0.92)	50.5 (1.73)	42.9 (2.20)	51.9 (3.48)							
Middle school	18.2 (0.67)	16.5 (1.26)	19.1 (1.78)	18.0 (2.66)							
High school	24.2 (0.76)	23.0 (1.46)	23.1 (1.77)	20.8 (2.68)							
≥ University	16.4 (0.72)	10.1 (1.06)	14.9 (1.76)	9.3 (2.11)							

Values are presented as weighted percentages (standard error). P-values were determined using analysis of variance or the Rao-Scott chi-squared test, as appropriate. Proportional differences in pairwise group comparisons are presented in Supplementary file Table 1.

Table 2 shows clinical, health behavior, perceived health status, and nutritional characteristics of the participants. Mean body mass index (BMI) and waist circumference (WC) were highest in the non-smoking/drinking group (BMI: 24.24 ± 0.13 kg/m^2^, p<0.001; WC: 88.56 ± 0.36 cm, p<0.001). However, low-density lipoprotein (LDL) was highest and high-density lipoprotein (HDL) was lowest in the smoking/non-drinking group (LDL: 108.39 ± 1.25 mg/dL, p=0.002; HDL: 44.01 ± 0.39 mg/dL, p<0.001), and triglycerides were highest in the smoking/drinking group (167.24 ± 7.12 mg/dL, p<0.001).

**Table 2:** Clinical characteristics, health behaviors, perceived health status, and nutritional characteristics of male participants, aged ≥65 years, according to smoking and drinking status, KNHANES 2007–2019

*Characteristics*	*Non-smoking/* *non-drinking* *(1)*	*Smoking/* *non-drinking* *(2)*	*Non-smoking/* *drinking* *(3)*	*Smoking/* *drinking* *(4)*	*p*	*p by multiple comparison*					
						*(1) vs (2)*	*(1) vs (3)*	*(1) vs (4)*	*(2) vs (3)*	*(2) vs (4)*	*(3) vs (4)*
**Total**, n (weighted n)	4953 (1809172)	1286 (455994)	730 (279532)	290 (110359)							
**Clinical details**											
SBP (mmHg)	126.74 ± 0.29	124.98 ± 0.62	131.04 ± 0.75	128.95 ± 1.18	<0.001	0.008	<0.001	0.065	<0.001	0.003	0.138
DBP (mmHg)	72.80 ± 0.18	70.90 ± 0.34	75.41 ± 0.45	74.04 ± 0.75	<0.001	<0.001	<0.001	0.112	<0.001	<0.001	0.119
Body mass index (kg/m^2^)	23.54 ± 0.05	22.81 ± 0.11	24.24 ± 0.13	23.09 ± 0.20	<0.001	<0.001	<0.001	0.026	<0.001	0.196	<0.001
Body mass index ≥25 kg/m^2^	29.9 (0.80)	22.0 (1.38)	38.4 (2.03)	23.6 (2.81)	<0.001	<0.001	<0.001	0.042	<0.001	0.593	<0.001
Waist circumference (cm)	86.02 ± 0.15	84.49 ± 0.32	88.56 ± 0.36	85.96 ± 0.61	<0.001	<0.001	<0.001	0.919	<0.001	0.027	<0.001
Waist circumference ≥90 cm	32.8 (0.79)	28.4 (1.51)	43.1 (2.15)	38.5 (3.39)	<0.001	0.010	<0.001	0.090	<0.001	0.003	0.246
Total cholesterol (mg/dL)	178.56 ± 0.66	178.91 ± 1.41	183.97 ± 1.57	177.41 ± 2.80	0.012	0.818	0.001	0.695	0.018	0.631	0.042
LDL (mg/dL)	107.77 ± 0.59	108.39 ± 1.25	104.03 ± 1.48	99.15 ± 2.77	0.002	0.656	0.018	0.003	0.026	0.003	0.122
HDL (mg/dL)	45.69 ± 0.20	44.01 ± 0.39	51.56 ± 0.57	48.19 ± 0.85	<0.001	<0.001	<0.001	0.004	<0.001	<0.001	0.001
Triglycerides (mg/dL)	129.74 ± 1.45	137.37 ± 2.65	156.43 ± 5.02	167.24 ± 7.12	<0.001	0.012	<0.001	<0.001	<0.001	<0.001	0.208
Hemoglobin (g/dL)	14.40 ± 0.03	14.53 ± 0.05	14.79 ± 0.06	14.87 ± 0.08	<0.001	0.018	<0.001	<0.001	0.001	<0.001	0.434
Fasting blood sugar (mg/dL)	107.02 ± 0.49	106.65 ± 1.02	111.67 ± 1.22	106.90 ± 1.53	0.004	0.736	<0.001	0.943	0.002	0.887	0.015
Hypertension	57.0 (0.83)	52.0 (1.68)	69.7 (1.95)	58.5 (3.37)	<0.001	0.008	<0.001	0.665	<0.001	0.077	0.003
Diabetes mellitus	25.3 (0.78)	27.3 (1.60)	29.8 (2.08)	24.7 (2.99)	0.138	0.252	0.035	0.842	0.340	0.429	0.162
Cardiovascular diseases	9.4 (0.51)	7.6 (0.88)	6.4 (1.03)	6.6 (1.44)	0.025	0.086	0.021	0.118	0.384	0.595	0.871
Cancer	11.0 (0.53)	6.0 (0.80)	8.1 (1.33)	1.2 (0.58)	<0.001	<0.001	0.069	<0.001	0.160	<0.001	<0.001
Anemia	13.8 (0.61)	10.5 (1.02)	7.6 (1.11)	4.6 (1.29)	<0.001	0.009	<0.001	<0.001	0.066	0.003	0.120
Stroke	7.9 (0.47)	8.3 (0.97)	5.6 (1.01)	4.4 (1.49)	0.079	0.720	0.061	0.079	0.061	0.068	0.546
COPD or asthma	4.8 (0.39)	4.2 (0.69)	3.4 (0.82)	4.8 (1.64)	0.527	0.442	0.165	0.999	0.473	0.711	0.403
Musculoskeletal diseases	15.6 (0.60)	13.3 (1.09)	14.2 (1.50)	14.3 (2.64)	0.343	0.068	0.392	0.651	0.607	0.700	0.968
Liver cirrhosis	1.0 (0.17)	0.6 (0.24)	0.6 (0.25)	0.4 (0.43)	0.373	0.236	0.224	0.386	0.917	0.747	0.792
Regular exercise	31.2 (0.85)	24.1 (1.49)	37.3 (2.16)	30.9 (3.36)	<0.001	<0.001	0.006	0.947	<0.001	0.055	0.126
Activity limitation	19.9 (0.68)	21.3 (1.38)	14.1 (1.50)	17.9 (2.67)	0.005	0.345	0.001	0.482	<0.001	0.267	0.189
**Perceived health status**					<0.001	<0.001	0.013	0.956	<0.001	0.206	0.190
Very good/good	30.4 (0.78)	24.7 (1.38)	35.3 (2.08)	29.5 (3.12)							
Fair	43.7 (0.87)	44.3 (1.75)	44.2 (2.21)	44.7 (3.41)							
Poor/very poor	25.9 (0.74)	31.1 (1.58)	20.6 (1.75)	25.8 (3.07)							
Perceived stress	12.2 (0.55)	16.6 (1.31)	9.7 (1.15)	13.5 (2.28)	<0.001	0.001	0.061	0.577	<0.001	0.262	0.095
Depressive mood	8.4 (0.47)	10.9 (1.01)	8.1 (1.15)	14.0 (2.58)	0.006	0.014	0.835	0.009	0.076	0.214	0.018
Trauma history	5.6 (0.41)	7.2 (0.88)	7.1 (1.12)	7.4 (1.73)	0.180	0.072	0.193	0.251	0.910	0.912	0.857
**Skipping meals**											
Breakfast	3.0 (0.29)	6.7 (0.90)	3.7 (0.80)	10.9 (2.51)	<0.001	<0.001	0.345	<0.001	0.016	0.076	<0.001
Lunch	4.6 (0.38)	9.9 (1.10)	6.5 (1.14)	10.0 (1.99)	<0.001	<0.001	0.078	<0.001	0.035	0.976	0.104
Dinner	2.3 (0.26)	2.7 (0.49)	4.9 (1.08)	6.6 (1.70)	<0.001	0.509	0.003	<0.001	0.036	0.003	0.373
**Daily intake**											
Calories (kcal)	1873.10 ± 12.75	1820.20 ± 22.79	2165.74 ± 37.26	2064.59 ± 56.67	<0.001	0.033	<0.001	0.001	<0.001	<0.001	0.138
Carbohydrates (g)	326.39 ± 2.18	314.00 ± 4.02	322.70 ± 5.39	295.65 ± 8.17	<0.001	0.004	0.526	<0.001	0.193	0.044	0.004
Protein (g)	63.66 ± 0.54	59.27 ± 0.96	70.88 ± 1.54	63.16 ± 2.18	<0.001	<0.001	<0.001	0.825	<0.001	0.100	0.004
Fat (g)	29.89 ± 0.41	27.37 ± 0.70	33.80 ± 1.16	30.56 ± 1.80	<0.001	0.001	0.001	0.716	<0.001	0.098	0.128
Calcium (mg)	500.23 ± 6.32	434.14 ± 11.10	510.93 ± 13.97	444.21 ± 17.31	<0.001	<0.001	0.488	0.003	<0.001	0.625	0.002
Iron (mg)	14.80 ± 0.18	13.83 ± 0.36	15.46 ± 0.43	14.35 ± 0.70	0.021	0.012	0.151	0.543	0.004	0.508	0.185
Sodium (mg)	3864.79 ± 42.41	3863.07 ± 85.20	4333.82 ± 115.91	4081.08 ± 172.69	0.001	0.985	<0.001	0.224	<0.001	0.250	0.228
Sodium ≥2000 mg	80.0 (0.67)	76.3 (1.50)	83.6 (1.58)	80.2 (2.63)	0.008	0.018	0.044	0.939	0.001	0.219	0.240
**Energy contribution** (%)											
Carbohydrate	70.71 ± 0.18	70.12 ± 0.40	61.93 ± 0.66	60.20 ± 1.12	<0.001	0.168	<0.001	<0.001	<0.001	<0.001	0.188
Protein	13.50 ± 0.07	12.94 ± 0.12	13.02 ± 0.16	12.20 ± 0.24	<0.001	<0.001	0.005	<0.001	0.704	0.007	0.004
Fat	13.71 ± 0.12	12.99 ± 0.24	13.43 ± 0.32	12.50 ± 0.51	0.007	0.007	0.412	0.021	0.274	0.390	0.119

Values are weighted mean ± SE for continuous variables or weighted percentage (SE) for categorical variables. SE: standard error. P-values were determined using analysis of variance or the Rao-Scott chi-squared test, as appropriate. Cardiovascular diseases were defined as a medical diagnosis of angina or myocardial infarction, while musculoskeletal diseases were defined as a medical diagnosis of arthritis, osteoarthritis, rheumatoid arthritis, or osteoporosis. COPD: chronic obstructive pulmonary disease. DBP: diastolic blood pressure. HDL: high-density lipoproteins. LDL: low-density lipoproteins. SBP: systolic blood pressure. Mean and proportion differences in pairwise group comparisons are presented in Supplementary file Table 2.

Considering comorbidities, the proportion of cardiovascular diseases (i.e. angina and/or myocardial infarction) was highest in the non-smoking/non-drinking group (9.4 ± 0.51%, p=0.025) and lowest in the non-smoking/drinking group (6.4 ± 1.03%, p=0.025). The proportion of cancer was also highest in the non-smoking/non-drinking group (11.0 ± 0.53%, p<0.001). Proportions of hypertension (69.7±1.95%, p<0.001) was highest in the non-smoking/drinking group.

The proportion of participation in regular exercise was highest in the non-smoking/drinking group (37.3 ± 2.16%, p<0.001) and lowest in the smoking/nondrinking group (24.1 ± 1.49%, p<0.001). Considering perceived health status, responses stating that health status was very good or good were highest in the non-smoking/drinking group (35.3 ± 2.08%, p<0.001); responses stating poor or very poor were highest in the smoking/non-drinking group (31.1 ± 1.58%, p<0.001). The proportion of perceived psychological stress was the highest in the smoking/non-drinking group (16.6 ± 1.31%, p<0.001) and lowest in the non-smoking/drinking group (9.7 ± 1.15%, p<0.001). The proportion of participants stating a depressive mood was also highest in the smoking/non-drinking group (14.0 ± 2.58%, p=0.006) and lowest in the non-smoking/drinking group (8.1 ± 1.15%, p=0.006). The proportion of skipped meals was highest in the smoking/drinking group and lowest in the non-smoking/non-drinking group.

### Smoking and/or drinking compared to non-smoking/non-drinking

Table 3 presents the factors associated with smoking and/or drinking behaviors in each group, using the non-smoking/non-drinking group as a reference.

**Table 3:** Factors associated with smoking and drinking of male participants, aged ≥65 years, according to smoking and drinking status, KNHANES 2007–2019

*Variables*	*Smoking/non-drinking*			*Non-smoking/drinking*			*Smoking/drinking*		
	*AOR (95% CI)*	*p*	*VIF*	*AOR (95% CI)*	*p*	*VIF*	*AOR (95% CI)*	*p*	*VIF*
**Age** (years)									
65–74	1.13 (0.92–1.40)	0.246	1.1372	1.53 (1.17–2.00)	0.002	1.1276	2.20 (1.35–3.61)	0.002	1.0891
≥75 ®	1			1			1		
**Marital status**									
Married ®	1			1			1		
Separated/divorced/widowed	1.55 (1.05–2.30)	0.029	1.9205	1.07 (0.60–1.90)	0.829	1.7295	0.82 (0.39–1.72)	0.602	2.1140
**Cohabitation**									
Living alone	1.13 (0.76–1.68)	0.534	1.9358	1.06 (0.59–1.89)	0.851	1.6950	1.67 (0.85–3.28)	0.134	2.0604
Living with family or others ®	1			1			1		
**Residence**									
Urban ®	1			1			1		
Rural	1.05 (0.86–1.29)	0.631	1.2080	0.99 (0.75–1.32)	0.962	1.1935	0.69 (0.47–1.01)	0.059	1.1990
**Currently employed**									
Yes ®	1			1			1		
No	0.64 (0.51–0.79)	<0.001	1.2787	1.20 (0.94–1.53)	0.137	1.2388	1.06 (0.72–1.56)	0.772	1.2579
**Household income**									
1st (highest) ®	1			1			1		
2nd	1.02 (0.78–1.35)	0.863	1.7370	0.78 (0.55–1.10)	0.157	1.5511	0.96 (0.55–1.67)	0.890	1.7513
3rd	1.04 (0.79–1.38)	0.772	1.8299	1.13 (0.79–1.61)	0.497	1.6663	1.31 (0.77–2.23)	0.324	1.9305
4th (lowest)	1.22 (0.91–1.63)	0.176	1.9671	0.93 (0.63–1.38)	0.731	1.7621	1.36 (0.79–2.34)	0.274	2.1871
**Types of health insurance**									
National health insurance ®	1			1			1		
Government medical aids for low income	1.45 (0.95–2.21)	0.087	1.1392	1.14 (0.60–2.17)	0.694	1.1059	1.79 (0.80–4.00)	0.154	1.2475
**Education level**									
≤ Elementary school	1.53 (1.08–2.16)	0.016	3.6239	1.27 (0.84–1.93)	0.256	3.2914	1.91 (0.91–4.01)	0.087	4.5658
Middle school	1.30 (0.91–1.86)	0.153	2.5258	1.21 (0.77–1.91)	0.412	2.3524	1.57 (0.72–3.42)	0.254	2.9016
High school	1.43 (1.01–2.03)	0.043	2.6307	0.99 (0.65–1.52)	0.974	2.4001	1.65 (0.77–3.52)	0.198	3.3644
≥ University ®	1			1			1		
**Clinical details**									
SBP (mmHg), per 10-unit increase	0.92 (0.87–0.98)	0.006	1.3149	1.05 (0.97–1.14)	0.194	1.2926	1.06 (0.94–1.19)	0.349	1.3004
Body mass index ≥25 kg/m^2^	0.59 (0.48–0.74)	<0.001	1.0926	1.44 (1.12–1.83)	0.004	1.1218	0.78 (0.51–1.19)	0.253	1.1132
Total cholesterol (mg/dL), per 10-unit increase	1.01 (0.98–1.04)	0.529	1.3082	0.97 (0.93–1.01)	0.110	1.2937	0.93 (0.88–0.98)	0.011	1.2915
HDL (mg/dL), per 10-unit increase	0.83 (0.75–0.92)	<0.001	1.3995	1.49 (1.35–1.64)	<0.001	1.4094	1.21 (1.04–1.42)	0.015	1.4238
Triglycerides (mg/dL), per 10-unit increase	1.00 (0.99–1.01)	0.978	1.3995	1.03 (1.02–1.05)	<0.001	1.3646	1.03 (1.01–1.05)	<0.001	1.3760
Hemoglobin (g/dL)	1.04 (0.95–1.14)	0.373	1.8839	1.10 (0.97–1.23)	0.134	1.7236	1.11 (0.94–1.31)	0.216	1.4770
FBS (mg/dL), per 10-unit increase	0.99 (0.96–1.03)	0.682	1.0704	1.04 (1.00–1.08)	0.076	1.0748	0.97 (0.91–1.03)	0.323	1.0885
Hypertension	0.95 (0.77–1.16)	0.617	1.3598	1.40 (1.06–1.87)	0.020	1.3407	0.94 (0.63–1.41)	0.770	1.3595
Cardiovascular diseases	0.83 (0.59–1.16)	0.273	1.0544	0.87 (0.54–1.41)	0.571	1.0548	0.97 (0.51–1.84)	0.932	1.0779
Cancer	0.63 (0.43–0.90)	0.012	1.0249	0.97 (0.65–1.45)	0.892	1.0349	0.09 (0.03–0.33)	<0.001	1.0205
Anemia	0.80 (0.55–1.16)	0.238	1.8007	0.86 (0.51–1.44)	0.566	1.6404	0.46 (0.19–1.10)	0.081	1.4162
Stroke	1.11 (0.75–1.64)	0.608	1.0504	0.98 (0.58–1.63)	0.925	1.0598	0.91 (0.38–2.19)	0.834	1.0721
Activity limitation	0.77 (0.60–0.99)	0.046	1.2688	0.87 (0.63–1.21)	0.417	1.2129	1.05 (0.67–1.64)	0.835	1.2671
No participation of regular exercise	1.31 (1.06–1.61)	0.011	1.0384	0.96 (0.74–1.24)	0.736	1.0603	1.04 (0.70–1.56)	0.838	1.0749
**Perceived health status**									
Very good/good ®	1			1			1		
Fair	1.27 (1.02–1.58)	0.032	1.4960	0.98 (0.75–1.27)	0.856	1.2940	0.90 (0.60–1.36)	0.629	1.3919
Poor/very poor	1.56 (1.21–2.00)	<0.001	1.8044	0.81 (0.57–1.15)	0.245	1.5442	0.89 (0.55–1.45)	0.647	1.6198
Perceived psychological stress	1.18 (0.90–1.55)	0.241	1.1400	0.80 (0.55–1.17)	0.245	1.1349	0.85 (0.48–1.49)	0.567	1.1554
Depressive mood	1.17 (0.88–1.57)	0.282	1.1511	1.31 (0.87–1.98)	0.192	1.1562	2.10 (1.20–3.67)	0.009	1.2238
**Skipping meals**									
Breakfast	1.90 (1.22–2.98)	0.005	1.0656	1.14 (0.59–2.21)	0.690	1.0553	2.09 (0.91–4.83)	0.084	1.0881
Lunch	1.68 (1.17–2.43)	0.005	1.0691	1.24 (0.72–2.14)	0.431	1.0526	1.20 (0.63–2.29)	0.587	1.1024
Dinner	0.96 (0.58–1.57)	0.858	1.0730	1.06 (0.47–2.40)	0.887	1.0633	0.93 (0.39–2.24)	0.870	1.1049
**Daily intake**									
Carbohydrates (g), per 100-unit increase	1.12 (0.97–1.29)	0.108	3.2404	1.14 (0.95–1.36)	0.157	2.7261	1.11 (0.84–1.45)	0.467	2.5268
Protein (g), per 10-unit increase	0.93 (0.87–0.99)	0.021	3.9428	0.90 (0.85–0.96)	<0.001	3.3208	0.84 (0.77–0.92)	<0.001	3.8636
Fat (g), per 10-unit increase	0.98 (0.87-1.12)	0.793	7.6203	1.20 (1.03–1.40)	0.019	7.0685	1.27 (1.07–1.51)	0.007	5.9733
Calcium (mg), per 100-unit increase	0.96 (0.92–1.01)	0.133	1.7015	0.99 (0.95–1.03)	0.734	1.6396	0.99 (0.93–1.04)	0.609	1.6761
Iron (mg), per 10-unit increase	1.01 (0.92–1.12)	0.790	1.5186	1.05 (0.95–1.16)	0.372	1.5410	1.09 (0.94–1.26)	0.237	1.4035
Sodium ≥2000 mg	0.94 (0.73–1.21)	0.624	1.2586	1.09 (0.76–1.57)	0.641	1.1877	1.39 (0.79–2.46)	0.255	1.2331
**Energy contribution** (%)									
Carbohydrates per 10-unit increase	0.70 (0.61–0.81)	<0.001	3.0787	0.44 (0.37–0.51)	<0.001	2.4347	0.38 (0.32–0.46)	<0.001	2.2280
Fat per 10-unit increase	0.77 (0.56–1.06)	0.111	5.8978	0.27 (0.18–0.43)	<0.001	5.1085	0.20 (0.12–0.34)	<0.001	3.9634

AOR: adjusted odds ratio. Results by multinomial logistic regression analysis adjusted for age, marital status, types of co-habitant, residence, employment status, household income, types of health insurance, education level, systolic blood pressure, body mass index (≥25 kg/m^2^), total cholesterol, high-density lipoprotein cholesterol, triglycerides, hemoglobin, fasting blood glucose, hypertension, cardiovascular disease, cancer, anemia, stroke, activity limitation, regular exercise, perceived health status, perceived stress, depressive mood, skipping meals (breakfast, lunch, and dinner), daily intake of macronutrients and minerals (carbohydrates, protein, fat, calcium, iron, and sodium (≥2000 mg), and energy contribution of carbohydrates, and fat. FBS: fasting blood sugar. HDL: high-density lipoprotein. SBP: systolic blood pressure. The crude ORs for Table 3 are presented in Supplementary file Table 3. VIF: variance inflation factor. Reference group: non-smoking/non-drinking group. ® Reference categories.

#### Factors associated with the smoking/non-drinking group

Being separated/divorced/widowed, an elementary or high school graduate, not participating in regular exercise, a perceived health status as fair and poor/very poor, and skipping breakfast or lunch were all significantly positively associated with smoking/non-drinking. Specifically, individuals who were separated/divorced/widowed were 1.55 times more likely [adjusted odds ratio (AOR)] to smoke and not drink (95% CI: 1.05–2.30, p=0.029), while elementary school graduates were 1.53 times more likely to smoke and not drink than university graduates (95% CI: 1.08–2.16, p=0.016).

This group was 1.31 times (95% CI: 1.06–1.61, p=0.011) more likely to not participate in regular exercise compared to the non-smoking/non-drinking group. Perceived health status as fair and poor/very poor was 1.27 times (95% CI: 1.02–1.58, p=0.032) and 1.56 times (95% CI: 1.21–2.00, p<0.001) more likely to be associated with the smoking/non-drinking group, respectively. Also, skipping breakfast and lunch were positively associated with this group, and participants were 1.90 times (95% CI: 1.22–2.98, p=0.005) and 1.68 times (95% CI: 1.17–2.43, p=0.005) more likely to fall into this group than other groups, respectively. In contrast, higher systolic blood pressure (SBP), BMI, and HDL levels, a history of cancer diagnosis, and a greater energy contribution from carbohydrates, were significantly and negatively associated with the smoking/non-drinking group, indicating that these factors were less likely to be observed in this group than in the reference group (Table 3).

#### Factors associated with the non-smoking/drinking group

Age (65–74 years compared to ≥75 years), BMI (≥25 kg/m^2^), elevated HDL and triglycerides, more daily fat intake and hypertension were the significant factors positively associated with participants in the non-smoking/drinking group. Specifically, the 65–74 years group was 1.53 times more likely to be associated with the non-smoking/drinking group compared to the ≥75 years group (95% CI: 1.17–2.00, p=0.002). In addition, those with BMI (≥25 kg/m^2^) and higher triglycerides (per 10 mg/dL increase) were 1.44 times (95% CI: 1.12–1.83, p=0.004) and 1.03 times (95% CI: 1.02–1.05, p<0.001) more likely to be associated with the non-smoking/drinking group, respectively. Having hypertension and more daily fat intake were 1.40 times (95% CI: 1.06–1.87, p=0.020) and 1.20 times (95% CI: 1.03–1.40, p=0.019) more likely to belong to this group, respectively (Table 3).

#### Factors associated with the smoking/drinking group

Age (65–74 years compared to ≥75 years), having higher triglyceride levels, more daily fat intake and depressive mood were significantly positively associated with the smoking/drinking group. However, being diagnosed with cancer was negatively associated with this group. Specifically, the 65–74 years group was 2.20 times more likely to be associated with the non-smoking/drinking group compared to the ≥75 years group (95% CI: 1.35–3.61, p=0.002); and having higher triglycerides and depressive mood were positively associated with the group, at 1.03 times (95% CI: 1.01–1.05, p<0.001) and 2.10 times (95% CI: 1.20–3.67, p=0.009), respectively. More daily fat intake was 1.27 times (95% CI: 1.07–1. 51, p=0.007) more likely associated with this group. Being diagnosed with cancer was found to be 0.09 times less likely to be associated with participants in this group (95% CI: 0.03–0.33, p<0.001) (Table 3).

## DISCUSSION

When we examined the annual changes in drinking and smoking rates of KNHANES participants over the past 13 years, the non-smoking/non-drinking group consistently accounted for the largest proportion, whereas the smoking/non-drinking and non-smoking/drinking groups showed an increasing trend in prevalence in recent years.

When examining the sociodemographic status of the study participants, the non-smoking/non-drinking group comprised the highest proportion of those aged ≥75 years and had the highest rates of marriage, living with family, university graduation, and highest household income. This suggests that this group has a better sociodemographic and economic status. Additionally, the non-smoking/drinking group also tended to have relatively better sociodemographic and economic statuses compared to both smoking groups, indicating that members of both smoking groups had relatively poorer sociodemographic and economic status. These findings are consistent with studies showing a higher prevalence of smokers among individuals with low sociodemographic and economic status^19,20^. In addition, when the groups were divided based on alcohol consumption (both drinking groups vs both non-drinking groups), no noticeable differences in sociodemographic or economic status were observed. We subsequently investigated clinical and health behaviors, perceived health status, and nutritional characteristics of the participants. The prevalence of comorbidities such as cardiovascular diseases and cancer was highest in the non-smoking/non-drinking group. This may imply that many people with illnesses quit smoking and drinking in this group. Hypertension and diabetes were highest in the non-smoking/drinking group, which can be explained by the higher average BMI and WC observed in this group.

Regarding examinations of participant health perceptions, psychological stress, and health behaviors, the non-smoking/drinking group had the least psychological stress and feelings of depression, felt subjectively healthier, and exercised the most regularly among the groups. It is not clear whether drinking has a direct influence on these findings, whether better physical and mental health lead to more drinking, or if other factors are involved. Individuals with higher education and income levels are more likely to consume alcohol and engage in risky drinking behavior^21,22^. In addition, certain cultures may interpret heavy drinking as a symbol of success or social status^23^. The ‘Alcohol Harm Paradox’ describes a situation where socioeconomically deprived groups experience more alcohol-related harm despite drinking less alcohol. This phenomenon indicates that even lower alcohol consumption can lead to more serious health impacts among disadvantaged populations, likely due to factors such as limited access to healthcare, social support, and poor overall health conditions^23^. Although the findings from the non-smoking/drinking group can be interpreted within this context, further research is needed to explore these associations thoroughly.

The smoking/non-drinking group had opposing findings to the non-smoking/drinking group, with the participants reporting the highest stress levels, high rates of feelings of depression, feeling unhealthy, and being the least active. Our results may infer that negative thoughts make it more difficult to quit smoking, but motivate the participants to abstain from alcohol because of feelings of guilt. This is supported by studies showing that unhealthy behaviors such as alcohol consumption and smoking can induce feelings of guilt^24-26^.

Regarding the significant factors in each group analyzed by multinomial logistic regression with the non-smoking/non-drinking group as the reference, low socioeconomic status, perceived poor health, unhealthy behavior such as skipping meals, and limited physical activity emerged as significant factors in the smoking/non-drinking group. Additionally, cancer diagnosis was negatively associated with the smoking/non-drinking group.

Aged 65–74 years (compared with ≥75 years), higher triglyceride levels, greater daily fat intake, and feelings of depression were all positively associated with the smoking/drinking group. In contrast, a cancer diagnosis was negatively associated with being in this group.

Perceived health status as poor and experiencing depressive moods were positively associated in the smoking/non-drinking group and the smoking/drinking group, respectively, implicating increased negative emotions in both smoking groups, as previously reported^27,28^. A history of cancer was a common negatively associated factor between groups, perhaps because a cancer diagnosis may motivate individuals to quit smoking. In the smoking/non-drinking group, skipping meals was positively associated, and skipping breakfast also tended to be positively associated with the smoking/drinking group. Smokers often engage in other unhealthy behaviors alongside smoking, creating a cluster of risk factors for various health problems^29,30^. Skipping meals could be considered an unhealthy behavior; thus, the tendency to have irregular meals in both smoking groups can be understood from this perspective.

In addition, looking at the factors associated with the non-smoking/drinking group, being aged 65–74 years compared to ≥75 years, higher triglycerides, and more daily fat intake were positively associated factors. Therefore, in both drinking groups (non-smoking/drinking and smoking/drinking groups), being 65–74 years, having higher triglyceride levels, and more daily fat intake were common associated factors. Skipping meals could be considered an unhealthy behavior; thus, the tendency to have irregular meals in both smoking groups can be understood from this perspective.

In summary, when examining the results of investigating the factors associated with each group, being relatively young and having metabolic changes, such as higher triglyceride levels and more daily fat intake, were commonly observed in both drinking groups. Conversely, having negative emotions was a common associated factor in both smoking groups. Further, unhealthy behaviors such as skipping meals tended to be associated with both smoking groups. In addition, both smoking groups tended to have low sociodemographic and economic statuses. The non-smoking/drinking group had the least psychological stress and feelings of depression, felt subjectively healthier, and exercised the most regularly among the groups.

Our study suggests that when promoting smoking cessation, it may be effective to concurrently address and modify smokers’ negative emotions. When encouraging alcohol abstinence, it is advisable to emphasize the increased likelihood of metabolic disorders.

There have been several studies investigating the factors influencing smoking and drinking among elderly men. Prior researchers have reported that factors affecting smoking and drinking in this population include the duration of addiction^12^, coping mechanisms for poor health or stress^31-32^, specific sociodemographic characteristics such as loneliness^33^, and economic factors such as low household income^34^. Our study findings somewhat align with these earlier studies. However, while past research has typically examined smoking and drinking separately, our study aimed to explore the differences between the two by dividing participants into four groups according to their smoking and drinking status, in order to compare the factors influencing both behaviors.

### Limitations

Our study findings are intriguing, but there are limitations in interpreting these results. Because our study was cross-sectional, we could not determine whether the observed conditions were the cause or the result. For example, regarding the non-smoking/non-drinking group having the highest prevalence of cardiovascular diseases and cancer, it is reasonable to believe that the occurrence of the disease led to smoking and drinking cessation, but this has not yet been proven. Additionally, a significant portion of the data in KNHANES is self-reported, which may reduce accuracy, and there may be other unmeasured confounding variables that could influence the results. Another limitation is the limited generalizability of the study findings, as the data were collected only from elderly men in a specific region. Furthermore, no interventions have been implemented to further support or validate these findings. Therefore, caution should be exercised when interpreting the results of this study.

## CONCLUSIONS

When comparing groups divided by smoking and alcohol consumption, metabolic changes such as elevated triglyceride levels were more common in the drinking groups than in the smoking groups, whereas negative emotions were more common in the smoking groups than in the drinking groups.

## Supplementary Material



## Data Availability

The data supporting this research are available from the authors on reasonable request.
