# Supplementary material for: Secondary cross-sectional analysis of smoking and drinking factors among older Korean men: A 13-year national survey
Source: Tob Induc Dis. 2025 Nov 21;23:10.18332/tid/211500. doi: 10.18332/tid/211500 (PMC12639415; doi:10.18332/tid/211500)
Supplement: Supplementary file 1 [file TID-23-181-s1.pdf]

Supplementary Table 1. Proportional differences in pairwise group comparisons of sociodemographic characteristics among male participants aged 65 years and older in KNHANES (2007–2019) by smoking and drinking status (N = 7,259)

	Proportion Difference (%) (95% CI)					
	(1) vs (2)	(1) vs (3)	(1) vs (4)	(2) vs (3)	(2) vs (4)	(3) vs (4)
Age group (years)	-5.6 (-9.0 – -2.1)	-14.6 (-18.3 – -10.9)	-21.6 (-26.8 – -16.5)	-9.1 (-13.6 – -4.6)	-16.1 (-21.9 – -10.2)	-7.0 (-13.1 – -0.9)
65–74	5.6 (2.1 - 9.0)	14.6 (10.9 - 18.3)	21.6 (16.5 - 26.8)	9.1 (4.6 - 13.6)	16.1 (10.2 - 21.9)	7.0 (0.9 - 13.1)
Marital status (%)						
Married	5.1 (2.6 - 7.7)	0.5 (-2.0 - 3.1)	4.0 (0.0 - 8.3)	-4.6 (-7.9 - -1.3)	-1.1 (-5.9 - 3.7)	3.5 (-1.4 - 8.3)
Separated/divorced/widowed	-5.1 (-7.7 - -2.6)	-0.5 (-3.1 - 2.0)	-4.0 (-8.3 - 0.0)	4.6 (1.3 - 7.9)	1.1 (-3.7 - 5.9)	-3.5 (-8.3 - 1.4)
Co-habitant type (%)						
Living alone	-4.3 (-6.4 - -2.2)	-1.3 (-3.7 - 1.2)	-6.1 (-10.2 - -2.0)	3.0 (0.2 - 5.9)	-1.8 (-6.2 - 2.6)	-4.8 (-9.3 - -0.3)
Living with family or others	4.3 (2.2 - 6.4)	1.3 (-1.2 - 3.7)	6.1 (2.0 - 10.2)	-3.0 (-5.9 - -0.2)	1.8 (-2.6 - 6.2)	4.8 (0.3 - 9.3)
Living area (%)						
Urban	5.4 (2.1 - 8.7)	0.7 (-3.3 - 4.7)	-2.3 (-7.5 - 3.0)	-4.7 (-9.4 - 0.1)	-7.7 (-13.3 - -2.0)	-3.0 (-9.0 - 3.1)
Rural	-5.4 (-8.7 - -2.1)	-0.7 (-4.7 - 3.3)	2.3 (-3.0 - 7.5)	4.7 (-0.1 - 9.4)	7.7 (2.0 - 13.3)	3.0 (-3.1 - 9.0)
Currently employed (%)						
Yes	-7.8 (-11.6 - -4.1)	-5.7 (-10.0 - -1.3)	-6.8 (-13.8 - 0.2)	2.2 (-3.0 - 7.4)	1.0 (-6.4 - 8.5)	-1.2 (-9.2 - 6.9)
No	7.8 (4.1 - 11.6)	5.7 (1.3 - 10.0)	6.8 (-0.2 - 13.8)	-2.2 (-7.4 - 3.0)	-1.0 (-8.5 - 6.4)	1.2 (-6.9 - 9.2)
Household income (Quartiles)						
1st (Highest)	5.5 (2.2 - 8.7)	-0.3 (-4.5 - 4.0)	6.5 (0.9 - 12.1)	-5.7 (-10.6 - -0.9)	1.0 (-5.2 - 7.2)	6.7 (-0.1 - 13.5)
2nd	2.2 (-0.9 - 5.4)	3.4 (-0.5 - 7.3)	3.2 (-2.8 - 9.3)	1.1 (-3.5 - 5.7)	1.0 (-5.6 - 7.6)	-0.1 (-6.9 - 6.7)
3rd	-2.1 (-5.1 - 1.0)	-2.9 (-7.0 - 1.2)	1.1 (-4.6 - 6.9)	-0.9 (-5.7 - 4.0)	3.2 (-2.9 - 9.4)	4.1 (-2.6 - 10.8)
4th (Lowest)	-5.6 (-8.9 - -2.3)	-0.2 (-3.9 - 3.6)	-10.9 (-17.3 - -4.4)	5.4 (0.9 - 10.0)	-5.3 (-12.1 - 1.6)	-10.7 (-17.9 - -3.5)
Types of health insurance (%)						

National health insurance	2.6 (1.0 - 4.2)	-0.8 (-2.5 - 0.8)	3.7 (0.1 - 7.6)	-3.4 (-5.4 - -1.5)	1.2 (-2.8 - 5.1)	4.6 (0.5 - 8.7)
Government medical aids for low income	-2.6 (-4.2 - -1.0)	0.8 (-0.8 - 2.5)	-3.7 (-7.6 - -0.1)	3.4 (1.5 - 5.4)	-1.2 (-5.1 - 2.8)	-4.6 (-8.7 - -0.5)
Education level (%)						
≤ Elementary school	-9.2 (-12.9 - -5.4)	-1.6 (-6.1 - 3.0)	-10.6 (-17.7 - -3.5)	7.6 (2.2 - 13.0)	-1.4 (-9.2 - 6.4)	-9.0 (-16.8 - -1.2)
Middle school	1.7 (-1.1 - 4.5)	-0.9 (-4.6 - 2.7)	0.2 (-5.1 - 5.5)	-2.6 (-7.0 - 1.7)	-1.5 (-7.3 - 4.3)	1.1 (-4.8 - 7.0)
High school	1.2 (-2.0 - 4.4)	1.0 (-2.8 - 4.9)	3.4 (-2.2 - 8.9)	-0.2 (-4.7 - 4.3)	2.1 (-4.0 - 8.3)	2.3 (-3.9 - 8.5)
≥ University	6.3 (3.8 - 8.7)	1.5 (-2.1 - 5.0)	7.0 (2.7 - 11.4)	-4.8 (-8.8 - -0.8)	0.8 (-3.9 - 5.4)	5.6 (-0.2 - 10.9)

Note: This table presents the proportion differences (%) and their 95% confidence intervals (CIs) corresponding to the analysis in Table 1 of the main manuscript. Groups (1) = Non-smoking / Non-drinking; (2) = Smoking / Non-drinking; (3) = Non-smoking / Drinking; (4) = Smoking / Drinking. The number of participants (weighted n) in each group: (1) = 4,953 (1,809,172); (2) = 1,286 (455,994); (3) = 730 (279, 532); (4) = 290 (110, 359).

Supplementary Table 2. Mean and proportion differences in pairwise group comparisons of clinical characteristics, health behaviours, perceived health status, and nutritional characteristics among male participants aged 65 years and older in the KNHANES (2007–2019) by smoking and drinking status (N = 7,259)

	Mean Difference (95% CI) or Proportion Difference (%) (95% CI)					
	(1) vs (2)	(1) vs (3)	(1) vs (4)	(2) vs (3)	(2) vs (4)	(3) vs (4)
SBP (mmHg)	1.8 (0.5 - 3.1)	-4.3 (-5.9 - -2.8)	-2.2 (-4.6 - 0.1)	-6.1 (-7.9 - -4.2)	-4.0 (-6.6 - -1.4)	2.1 (-0.7 - 4.8)
DBP (mmHg)	1.9 (1.2 - 2.6)	-2.6 (-3.5 - -1.7)	-1.2 (-2.8 - 0.3)	-4.5 (-5.6 - -3.4)	-3.1 (-4.7 - -1.6)	1.4 (-0.4 - 3.1)
Body mass index (kg/m^2)	0.7 (0.5 - 1.0)	-0.7 (-1.0 - -0.4)	0.5 (0.1 - 0.8)	-1.4 (-1.7 - -1.1)	-0.3 (-0.7 - 0.1)	1.1 (0.7 - 1.6)
Body mass index ≥ 25 kg/m^2	8.0 (4.8 - 11.1)	-8.4 (-12.6 - -4.2)	6.3 (0.6 - 12.1)	-16.4 (-21.1 - -11.6)	-1.6 (-7.7 - 4.4)	14.7 (8.1 - 21.4)
Waist circumference (cm)	1.5 (0.8 - 2.2)	-2.5 (-3.3 - -1.8)	0.1 (-1.2 - 1.3)	-4.1 (-5.0 - -3.2)	-1.5 (-2.8 - -0.2)	2.6 (1.2 - 4.0)
Waist circumference ≥ 90 cm	4.4 (1.1 - 7.6)	-10.3 (-14.7 - -5.9)	-5.7 (-12.4 - 1.1)	-14.7 (-19.8 - -9.6)	-10.0 (-17.0 - -3.1)	4.6 (-3.1 - 12.4)
Total cholesterol (mg/dL)	-0.4 (-3.4 - 2.6)	-5.4 (-8.7 - -2.1)	1.2 (-4.6 - 6.9)	-5.1 (-9.3 - -0.9)	1.5 (-4.6 - 7.6)	6.6 (0.2 - 12.9)
LDL (mg/dL)	-0.6 (-3.3 - 2.1)	3.7 (0.6 - 6.8)	8.6 (3.0 - 14.3)	4.4 (0.5 - 8.2)	9.2 (3.2 - 15.3)	4.9 (-1.3 - 11.1)
HDL (mg/dL)	1.7 (0.8 - 2.5)	-5.9 (-7.0 - -4.7)	-2.5 (-4.2 - -0.8)	-7.6 (-8.9 - -6.2)	-4.2 (-6.0 - -2.3)	3.4 (1.4 - 5.4)
Triglyceride (mg/dL)	-7.6 (-13.6 - -1.7)	-26.7 (-36.9 - -16.5)	-37.5 (-51.8 - -23.3)	-19.1 (-30.2 - -7.9)	-29.9 (-45.0 - -14.7)	-10.8 (-27.7 - 6.0)
Haemoglobin (g/dL)	-0.1 (-0.2 - -0.0)	-0.4 (-0.5 - -0.3)	-0.5 (-0.6 - -0.3)	-0.3 (-0.4 - -0.1)	-0.3 (-0.5 - -0.2)	-0.1 (-0.3 - 0.1)
Fasting blood sugar (mg/dL)	0.4 (-1.8 - 2.5)	-4.6 (-7.2 - -2.1)	0.1 (-3.1 - 3.3)	-5.0 (-8.2 - -1.9)	-0.3 (-3.8 - 3.3)	4.8 (0.9 - 8.6)
Hypertension (%)	5.0 (1.3 - 8.6)	-12.7 (-16.8 - -8.6)	-1.5 (-8.3 - 5.3)	-17.7 (-22.9 - -12.5)	-6.5 (-13.6 - 0.6)	11.2 (3.6 - 18.8)
Diabetes mellitus (%)	-2.0 (-5.5 - 1.5)	-4.5 (-8.9 - -0.2)	0.6 (-5.5 - 6.8)	-2.5 (-7.7 - 2.7)	2.7 (-3.8 - 9.1)	5.2 (-1.9 - 12.2)
Cardiovascular diseases (%)	1.8 (-0.1 - 3.8)	3.0 (0.8 - 5.3)	2.7 (-0.3 - 5.8)	1.2 (-1.5 - 3.9)	0.9 (-2.4 - 4.3)	-0.3 (-3.8 - 3.2)
Cancer (%)	5.0 (3.2 - 6.9)	3.0 (-0.1 - 5.8)	9.8 (8.3 - 11.3)	-2.1 (-5.1 - 1.0)	4.8 (2.9 - 6.6)	6.8 (4.0 - 9.7)
Anaemia (%)	3.3 (1.0 - 5.7)	6.2 (3.7 - 8.8)	9.2 (6.3 - 12.0)	2.9 (-0.1 - 5.9)	5.8 (2.7 - 9.0)	2.9 (-0.5 - 6.3)
Stroke (%)	-0.4 (-2.5 - 1.7)	2.4 (-0.2 - 4.6)	3.5 (-0.4 - 6.6)	2.7 (-0.0 - 5.5)	3.9 (-0.4 - 7.4)	1.1 (-2.4 - 4.7)
COPD or Asthma (%)	0.6 (-0.9 - 2.2)	1.4 (-0.4 - 3.2)	0.0 (-3.3 - 3.3)	0.8 (-1.3 - 2.9)	-0.6 (-4.1 - 2.9)	-1.4 (-5.0 - 2.2)
Musculoskeletal diseases (%)	2.3 (-0.1 - 4.8)	1.4 (-1.7 - 4.5)	1.3 (-4.1 - 6.6)	-0.9 (-4.6 - 2.7)	-1.1 (-6.6 - 4.5)	-0.1 (-6.2 - 6.0)

Liver cirrhosis (%)	0.4 (-0.2 - 1.0)	0.4 (-0.2 - 1.0)	0.6 (-0.3 - 1.5)	0.0 (-0.6 - 0.7)	0.2 (-0.8 - 1.1)	0.1 (-0.8 - 1.1)
Regular exercise (%)	7.1 (3.8 - 10.3)	-6.1 (-10.6 - -1.6)	0.2 (-6.5 - 6.9)	-13.1 (-18.3 - -8.0)	-6.8 (-14.1 - 0.5)	6.3 (-1.6 - 14.2)
Activity limitation (%)	-1.4 (-4.5 - 1.6)	5.8 (2.6 - 9.1)	2.0 (-3.4 - 7.4)	7.3 (3.3 - 11.2)	3.4 (-2.4 - 9.2)	-3.8 (-9.8 - 2.1)
Perceived health status (%)						
Very good/Good	5.7 (2.6 - 8.8)	-4.9 (-9.2 - -0.5)	0.9 (-5.5 - 7.2)	-10.6 (-15.4 - -5.7)	-4.8 (-11.4 - 1.7)	5.7 (-1.6 - 13.0)
Fair	-0.6 (-4.3 - 3.2)	-0.4 (-5.0 - 4.1)	-1.0 (-7.8 - 5.9)	0.1 (-5.3 - 5.6)	-0.4 (-7.8 - 7.0)	-0.5 (-8.6 - 7.6)
Poor/Very poor	-5.1 (-8.5 - -1.7)	5.3 (1.6 - 9.0)	0.1 (-6.1 - 6.3)	10.5 (6.0 - 15.0)	5.2 (-1.6 - 12.0)	-5.2 (-12.1 - 1.7)
Perceived stress (%)	-4.4 (-7.2 - -1.5)	2.5 (-0.1 - 5.0)	-1.3 (-5.9 - 3.3)	6.9 (3.4 - 10.3)	3.1 (-2.1 - 8.3)	-3.8 (-8.6 - 1.0)
Depressive mood (%)	-2.5 (-4.7 - -0.4)	0.3 (-2.2 - 2.7)	-5.6 (-10.8 - -0.5)	2.8 (-0.2 - 5.8)	-3.1 (-8.4 - 2.2)	-5.9 (-11.4 - -0.4)
Trauma history (%)	-1.6 (-3.5 - 0.3)	-1.4 (-3.8 - 0.9)	-1.8 (-5.3 - 1.7)	0.2 (-2.6 - 3.0)	-0.2 (-4.0 - 3.6)	-0.4 (-4.5 - 3.7)
Skipping meals						
Skipping breakfast (%)	-3.1 (-4.9 - -1.3)	-0.4 (-2.1 - 1.3)	-6.3 (-11.1 - -1.5)	2.7 (0.4 - 5.0)	-3.2 (-8.3 - 1.9)	-5.9 (-10.9 - -0.9)
Skipping lunch (%)	-4.6 (-6.8 - -2.4)	-1.6 (-4.1 - 0.8)	-5.7 (-9.9 - -1.5)	3.0 (0.1 - 6.0)	-1.1 (-5.8 - 3.7)	-4.0 (-8.8 - 0.7)
Skipping dinner (%)	-0.3 (-1.4 - 0.8)	-2.8 (-5.2 - -0.4)	-4.2 (-7.6 - -0.8)	-2.5 (-5.0 - 0.0)	-3.9 (-7.4 - -0.4)	-1.4 (-5.5 - 2.7)
Daily calorie intake (kcal)	52.7 (4.2 - 101.2)	-293.3 (-370.5 - -216.1)	-193.3 (-307.5 - -79.0)	-346.0 (-432.2 - -259.8)	-246.0 (-363.3 - -128.6)	100.0 (-33.5 - 233.5)
Daily carbohydrate intake (g)	12.3 (3.9 - 20.8)	3.7 (-7.7 - 15.1)	30.6 (14.0 - 47.1)	-8.6 (-21.7 - 4.4)	18.2 (0.4 - 36.0)	26.9 (8.3 - 45.4)
Daily protein intake (g)	4.4 (2.3 - 6.5)	-7.3 (-10.4 - -4.1)	0.4 (-4.0 - 4.9)	-11.6 (-15.2 - -8.1)	-4.0 (-8.6 - 0.7)	7.7 (2.5 - 12.9)
Daily fat intake (g)	2.5 (1.0 - 4.1)	-4.0 (-6.4 - -1.6)	-0.7 (-4.4 - 2.9)	-6.5 (-9.2 - -3.9)	-3.2 (-7.0 - 0.6)	3.3 (-0.9 - 7.5)
Daily calcium intake (mg)	64.7 (40.1 - 89.3)	-9.5 (-39.7 - 20.7)	55.8 (19.4 - 92.3)	-74.2 (-109.1 - -39.3)	-8.9 (-49.2 - 31.5)	65.3 (22.4 - 108.3)
Daily iron intake (mg)	1.0 (0.2 - 1.7)	-0.4 (-1.3 - 0.5)	0.4 (-1.0 - 1.9)	-1.4 (-2.5 - -0.3)	-0.5 (-2.1 - 1.0)	0.9 (-0.8 - 2.5)
Daily Na intake (mg)	-4.3 (-185.7 - 177.2)	-472.3 (-712.2 – -232.5)	-201.6 (-548.8 - 145.6)	-468.1 (-747.9 – -188.2)	-197.3 (-567.8 - 173.2)	270.7 (-139.9 - 681.4)
Daily Na intake (≥2,000 mg)	3.8 (0.6 - 7.1)	-3.8 (-7.2 - -0.4)	-0.1 (-5.5 - 5.3)	-7.6 (-12.0 - -3.2)	-3.9 (-10.0 - 2.1)	3.7 (-2.2 - 9.6)
Energy contribution (%)						

Carbohydrate	0.6 (-0.2 - 1.4)	8.8 (7.5 - 10.1)	10.5 (8.3 - 12.7)	8.2 (6.7 - 9.6)	9.9 (7.6 - 12.3)	1.7 (-0.8 - 4.3)
Protein	0.6 (0.3 - 0.8)	0.5 (0.1 - 0.8)	1.3 (0.8 - 1.8)	-0.1 (-0.5 - 0.3)	0.7 (0.2 - 1.3)	0.8 (0.2 - 1.4)
Fat	0.7 (0.2 - 1.2)	0.3 (-0.4 - 0.9)	1.2 (0.2 - 2.2)	-0.5 (-1.3 - 0.3)	0.5 (-0.6 - 1.6)	0.9 (-0.2 - 2.1)

Note: This table shows the mean and proportion differences (95% CIs) corresponding to the analysis in Table 2 of the main manuscript. Groups (1) = Non-smoking / Non-drinking; (2) = Smoking / Non-drinking; (3) = Non-smoking / Drinking; (4) = Smoking / Drinking. The number of participants (weighted n) in each group: (1) = 4,953 (1,809,172); (2) = 1,286 (455,994); (3) = 730 (279, 532); (4) = 290 (110, 359).

Cardiovascular diseases were defined as a medical diagnosis of angina or myocardial infarction, while musculoskeletal diseases were defined as a medical diagnosis of arthritis, osteoarthritis, rheumatoid arthritis, or osteoporosis. COPD, chronic obstructive pulmonary disease; DBP, diastolic blood pressure; HDL, high-density lipoproteins; LDL, low-density lipoproteins; Na, sodium. SBP, systolic blood pressure.

Supplementary Table 3. Factors associated with smoking and drinking among male participants aged 65 years and older in the KNHANES (2007–2019): Unadjusted odds ratios (ORs)

	Smoking / Non-drinking		Non-smoking / Drinking		Smoking / Drinking	
	Unadjusted		Unadjusted		Unadjusted	
	OR (95% CI)	*P*-value	OR (95% CI)	*P*-value	OR (95% CI)	*P*-value
Age group (year)						
65–74	1.28 (1.09-1.49)	0.0020	2.02 (1.65-2.46)	<.0001	3.15 (2.18-4.56)	<.0001
≥ 75	1 (ref)		1 (ref)		1 (ref)	
Marital status						
Married	1 (ref)		1 (ref)		1 (ref)	
Separated/divorced/widowed	1.63 (1.31-2.04)	<.0001	1.06 (0.80-1.42)	0.6860	1.49 (1.02-2.17)	0.0402
Co-habitant type						
Living alone	1.63 (1.32-2.01)	<.0001	1.18 (0.87-1.59)	0.2821	1.91 (1.34-2.72)	0.0004
Living with family or others	1 (ref)		1 (ref)		1 (ref)	
Living area						
Urban	1 (ref)		1 (ref)		1 (ref)	
Rural	1.31 (1.12-1.53)	0.0009	1.04 (0.84-1.28)	0.7294	0.88 (0.66-1.19)	0.4104
Currently employed						
Yes	1 (ref)		1 (ref)		1 (ref)	
No	0.72 (0.62-0.84)	<.0001	0.79 (0.66-0.94)	0.0092	0.75 (0.57-1.00)	0.0515
Household income						
1st (Highest)	1 (ref)		1 (ref)		1 (ref)	
2nd	1.14 (0.92-1.42)	0.2427	0.86 (0.66-1.12)	0.2736	1.14 (0.75-1.74)	0.5308
3rd	1.36 (1.10-1.68)	0.0047	1.11 (0.86-1.44)	0.4088	1.24 (0.82-1.88)	0.3034
4th (Lowest)	1.55 (1.25-1.92)	<.0001	1.00 (0.77-1.29)	0.9893	1.93 (1.32-2.82)	0.0007
Types of health insurance						
National health insurance	1 (ref)		1 (ref)		1 (ref)	

Government medical aids for low income	1.72 (1.28-2.30)	0.0003	0.78 (0.45-1.35)	0.3732	2.05 (1.16-3.64)	0.0140
Education level						
≤ Elementary school	1.98 (1.53-2.55)	<.0001	1.14 (0.85-1.53)	0.3882	2.20 (1.31-3.69)	0.0027
Middle school	1.47 (1.10-1.96)	0.0091	1.15 (0.82-1.62)	0.4075	1.73 (0.98-3.09)	0.0610
High school	1.54 (1.16-2.04)	0.0028	1.05 (0.76-1.45)	0.7642	1.51 (0.86-2.64)	0.1496
≥ University	1 (ref)		1 (ref)		1 (ref)	
SBP (mmHg), per 10-unit increase	0.94 (0.90-0.98)	0.0095	1.15 (1.10-1.21)	<.0001	1.08 (1.00-1.17)	0.0579
Body mass index (≥ 25 kg/m^2)	0.66 (0.55-0.79)	<.0001	1.46 (1.22-1.74)	<.0001	0.72 (0.53-0.99)	0.0437
Total cholesterol (mg/dL), per 10-unit increase	1.00 (0.98-1.03)	0.8180	1.04 (1.02-1.07)	0.0011	0.99 (0.95-1.04)	0.6964
HDL (mg/dL), per 10-unit increase	0.86 (0.80-0.93)	0.0002	1.49 (1.38-1.60)	<.0001	1.21 (1.07-1.36)	0.0020
Triglyceride (mg/dL), per 10-unit increase	1.01 (1.00-1.02)	0.0104	1.03 (1.02-1.04)	<.0001	1.04 (1.02-1.05)	<.0001
Haemoglobin (g/dL)	1.07 (1.01-1.13)	0.0193	1.23 (1.14-1.32)	<.0001	1.29 (1.17-1.42)	<.0001
FBS (mg/dL), per 10-unit increase	0.99 (0.96-1.03)	0.7399	1.05 (1.03-1.08)	0.0001	1.00 (0.95-1.04)	0.9429
Hypertension	0.82 (0.71-0.95)	0.0078	1.74 (1.43-2.10)	<.0001	1.06 (0.80-1.41)	0.6649
Cardiovascular diseases	0.79 (0.61-1.03)	0.0866	0.65 (0.46-0.94)	0.0224	0.69 (0.43-1.10)	0.1196
Cancer	0.52 (0.38-0.69)	<.0001	0.71 (0.49-1.03)	0.0706	0.10 (0.04-0.26)	<.0001
Anaemia	0.73 (0.58-0.92)	0.0092	0.51 (0.37-0.71)	<.0001	0.30 (0.17-0.54)	<.0001
Stroke	1.05 (0.79-1.40)	0.7198	0.69 (0.46-1.02)	0.0626	0.54 (0.27-1.09)	0.0836
Activity limitation	1.09 (0.91-1.31)	0.3458	0.66 (0.51-0.85)	0.0016	0.88 (0.61-1.26)	0.4824
No participation of regular exercise	1.42 (1.20-1.69)	<.0001	0.76 (0.63-0.93)	0.0063	1.01 (0.74-1.38)	0.9468
Perceived health status						
Very good/Good	1 (ref)		1 (ref)		1 (ref)	
Fair	1.25 (1.04-1.49)	0.0158	0.87 (0.71-1.07)	0.1917	1.05 (0.76-1.46)	0.7638
Poor/Very poor	1.47 (1.22-1.79)	<.0001	0.68 (0.53-0.88)	0.0030	1.02 (0.70-1.50)	0.8991
Perceived psychological stress	1.43 (1.15-1.77)	0.0011	0.77 (0.59-1.01)	0.0624	1.12 (0.75-1.66)	0.5772
Depressive mood	1.34 (1.06-1.69)	0.0141	0.97 (0.69-1.34)	0.8349	1.78 (1.15-2.76)	0.0097
Skipping breakfast	2.36 (1.67-3.33)	<.0001	1.26 (0.78-2.05)	0.3459	3.98 (2.33-6.80)	<.0001
Skipping lunch	2.28 (1.73-3.01)	<.0001	1.44 (0.96-2.16)	0.0792	2.30 (1.45-3.65)	0.0004
Skipping dinner	1.16 (0.75-1.77)	0.5091	2.14 (1.29-3.55)	0.0033	2.95 (1.65-5.26)	0.0003

Daily carbohydrate intake (g), per 100-unit increase	0.91 (0.86-0.97)	0.0061	0.97 (0.90-1.06)	0.5320	0.78 (0.67-0.91)	0.0013
Daily protein intake (g), per 10-unit increase	0.95 (0.92-0.98)	0.0001	1.07 (1.04-1.09)	<.0001	0.99 (0.95-1.04)	0.8273
Daily fat intake (g), per 10-unit increase	0.95 (0.91-0.98)	0.0033	1.06 (1.03-1.10)	0.0006	1.01 (0.95-1.08)	0.7065
Daily Calcium intake (mg), per 100-unit increase	0.93 (0.90-0.96)	<.0001	1.01 (0.99-1.03)	0.4773	0.94 (0.90-0.99)	0.0114
Daily Iron intake (mg), per 10-unit increase	0.90 (0.82-0.99)	0.0383	1.05 (0.99-1.11)	0.1299	0.96 (0.83-1.11)	0.5842
Daily Sodium (≥ 2,000 mg/day)	0.81 (0.67-0.96)	0.0187	1.28 (1.01-1.63)	0.0443	1.01 (0.72-1.42)	0.9387
Energy contribution						
Carbohydrate (%), per 10-unit increase	0.96 (0.90-1.02)	0.1619	0.59 (0.55-0.63)	<.0001	0.55 (0.50-0.61)	<.0001
Fat (%), per 10-unit increase	0.87 (0.79-0.97)	0.0086	0.95 (0.84-1.08)	0.4182	0.79 (0.63-0.98)	0.0328

Note: Unadjusted odds ratios (ORs) and 95% confidence intervals (CIs) were estimated using multinomial logistic regression, corresponding to the analysis presented in Table 3 of the main manuscript. The reference group was the non-smoking and non-drinking group. Abbreviation: ref = reference.

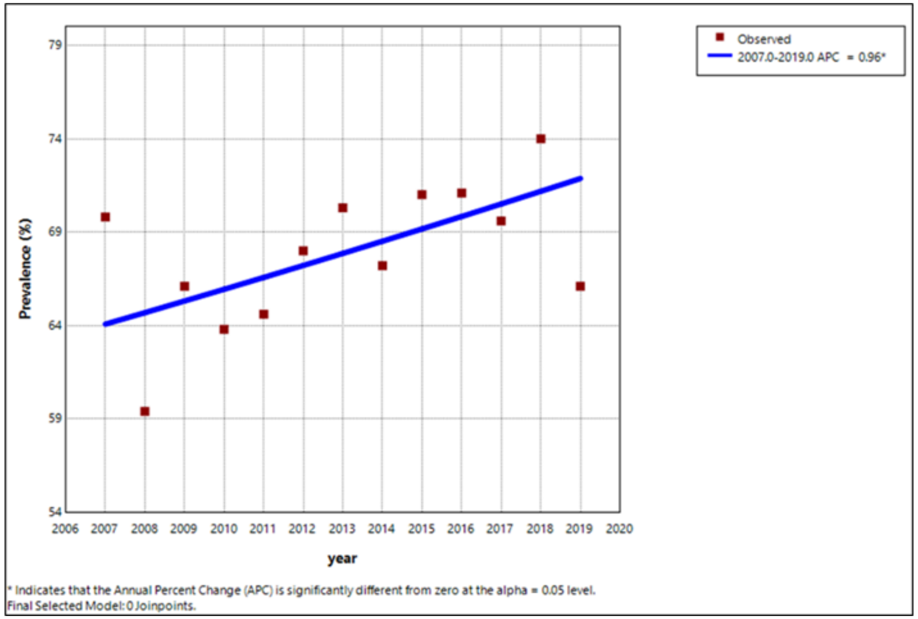

Supplementary Figure 1 (a)

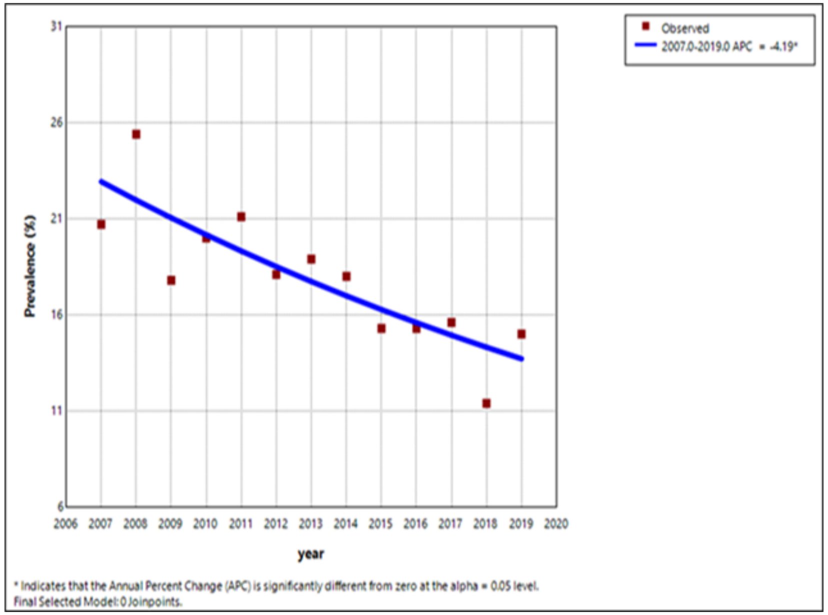

Supplementary Figure 1 (b)

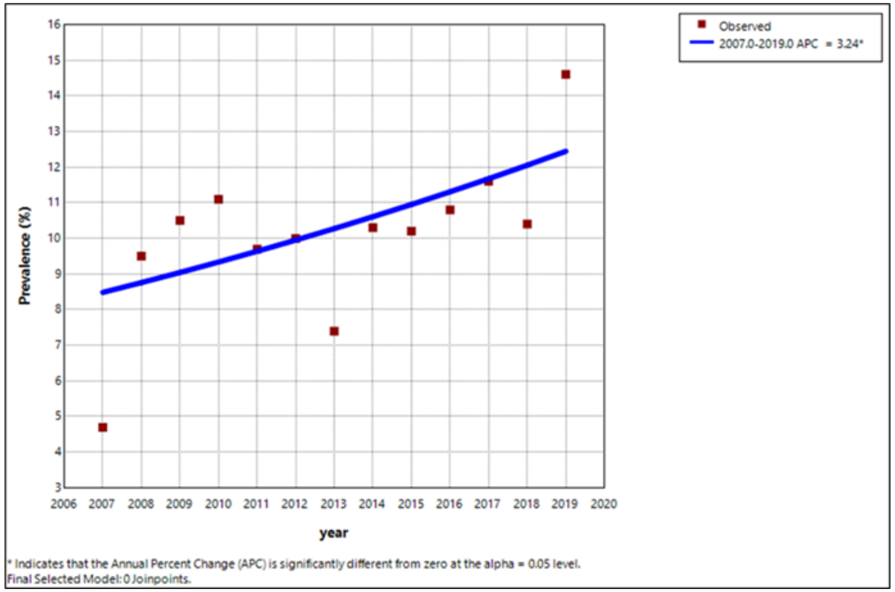

Supplementary Figure 1 (c)

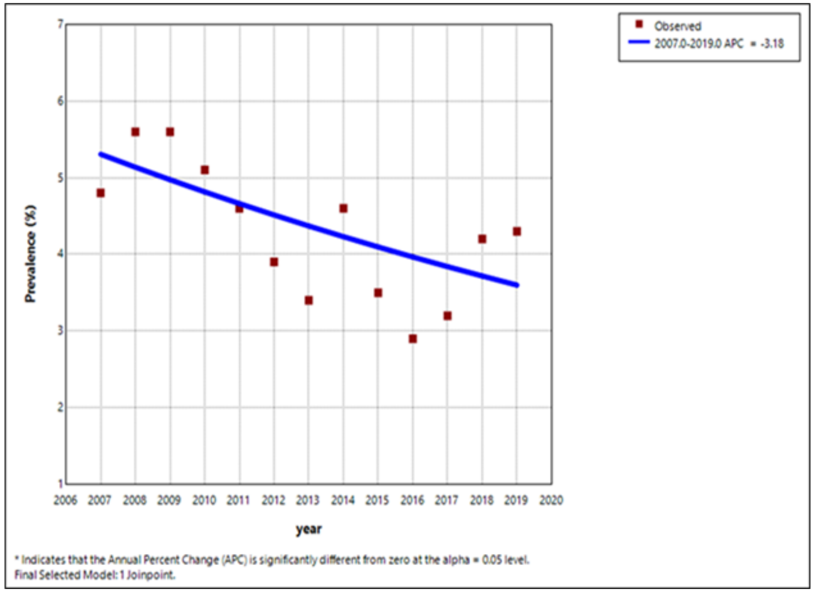

Supplementary Figure 1 (d)

Supplementary Figure 1**.** Joinpoint regression results for the prevalence of smoking and drinking among male participants aged 65 years and older in the KNHANES (2007–2019): (a) Non-smoking/Non-drinking, (b) Smoking/Non-drinking, (c) Non-smoking/Drinking, (d) Smoking/Drinking.

Supplementary Table 4. Joinpoint regression results corresponding to Supplementary Figure 1 (a–d)

Group	Lower Endpoint – Upper Endpoint (Years)	APC (%)	95% CI	*P*-Value
Non-smoking/Non-drinking (a)	2007- 2019	0.96	0.086 to 1.960	0.0312
Smoking/Non-drinking (b)	2007- 2019	-4.19	-6.425 to -2.132	< 0.0001
Non-smoking/Drinking (c)	2007- 2019	3.24	0.809 to 6.349	0.0072
Smoking/Drinking (d)	2007- 2019	-3.18	-6.986 to 0.474	0.0808

Abbreviations: APC = annual percentage change; CI = confidence interval.

© **2025 Lee J. et al.**
